# Selection of Probiotics for Honey Bees: The In Vitro Inhibition of *Paenibacillus larvae*, *Melissococcus plutonius*, and *Serratia marcescens* Strain Sicaria by Host-Specific Lactobacilli and Bifidobacteria

**DOI:** 10.3390/microorganisms13051159

**Published:** 2025-05-20

**Authors:** Buse Dengiz, Jiří Killer, Jaroslav Havlík, Pavel Dobeš, Pavel Hyršl

**Affiliations:** 1 Department of Food Science, Czech University of Life Sciences Prague, 165 00 Prague, Czech Republic; dengiz@af.czu.cz (B.D.); havlik@af.czu.cz (J.H.); 2 Laboratory of Anaerobic Microbiology, Institute of Animal Physiology and Genetics, Czech Academy of Sciences, 142 20 Prague, Czech Republic; 3 Department of Experimental Biology, Faculty of Science, Masaryk University, 625 00 Brno, Czech Republic; pavel.dobes@mail.muni.cz (P.D.); hyrsl@sci.muni.cz (P.H.)

**Keywords:** bifidobacteria, lactobacilli, in vitro inhibition, *Paenibacillus larvae*, *Melissococcus plutonius*, *Serratia marcescens* strain sicaria, honey bee probiotics

## Abstract

Host-specific *Lactobacillus* and *Bifidobacterium* species constitute the core microbiota of the honey bee digestive tract and are recognized for their probiotic properties. One of the properties of these bacteria is the inhibition of bacterial pathogens such as *Paenibacillus larvae* and *Melissococcus plutonius*, the causative agents of American and European foulbrood, respectively. Additionally, *Serratia marcescens* has emerged as a relevant opportunistic pathogen. Although several previously published studies have examined the inhibition of selected bacterial pathogens of bees by members of the bee physiological microbiota, none have simultaneously investigated the inhibition of multiple clinical isolates of *P. larvae*, *M. plutonius*, and *S. marcescens* using a wide range of bifidobacterial and lactobacilli strains isolated from various locations within a single country. Thus, this study evaluated the antimicrobial potential of *Lactobacillus* and *Bifidobacterium* strains against these pathogens, with a focus on strain-dependent inhibition. A total of 111 bacterial strains (62 *Lactobacillus* and 49 *Bifidobacterium*) were isolated from the digestive tracts of honey bees collected from eight sites across the Czech Republic. Using 16S rRNA gene sequencing, the isolates were classified and tested in vitro against four *P. larvae* isolates, one *M. plutonius* isolate, and the *S. marcescens* strain sicaria in modified BHI medium. Twenty-eight strains (~26%) exhibited strong inhibition (≥21 mm) against at least two *P. larvae* isolates, while 12 strains showed moderate inhibition (16–20 mm) against all four isolates. Inhibition of *M. plutonius* and *S. marcescens* was observed in three and twenty strains, respectively. The most effective strains belonged to *Bifidobacterium asteroides*, *B. choladohabitans*, *B. polysaccharolyticum*, *Lactobacillus apis*, *L. helsingborgensis*, *L. kullabergensis*, and *L. melliventris*. These results underscore the strain-dependent nature of antimicrobial activity and highlight the importance of selecting probiotic strains with broad-spectrum pathogen inhibition to support honey bee health.

## 1. Introduction

Interest in studying the host-specific microbiota of the digestive tracts of bees and other pollinators has significantly increased over the past decade, during which several bacterial species and genera have been described [1,2,3,4,5,6,7,8,9]. Their presence and activity in the gut are associated with numerous probiotic properties, most of which are attributed to host-specific *Lactobacillus* species, with some evidence also highlighting the probiotic potential of *Bifidobacterium* spp. Several species of gut-associated probiotics contribute to honey bee health through two distinct yet complementary mechanisms. First, they are capable of producing a variety of digestive enzymes, such as those that degrade amygdalin, arabinose, xylose, galactose, mannose, lactose, and raffinose, enhancing nutrient absorption and improving the bee’s ability to utilize diverse carbohydrate sources [10,11,12]. These enzymatic activities play a crucial role in breaking down complex polysaccharides, oligosaccharides, and peptides found in the bee diet. Second, some strains have demonstrated the ability to degrade xenobiotics, including harmful insecticides, thus potentially reducing chemical toxicity in the gut environment. This dual functionality, facilitating digestion and detoxification, highlights the potential of certain probiotic strains to enhance honey bee resilience to dietary and environmental stressors [13,14].

Other probiotic functions involve enhancing bee nutrition by synthesizing amino acids and vitamins [15,16], maintaining intestinal homeostasis, stimulating antimicrobial peptide expression [17,18,19], regulating hormone-driven weight gain [20], increasing brood and honey production [21], improving the longevity of queens and overwintering bees [22,23], preserving the nutritional value of bee food [24], mitigating antibiotic-induced dysbiosis [25,26], and even modulating learning [27,28] and memory behavior [29]. However, the most frequently cited probiotic effects are those involving the inhibition of prokaryotic [19,30,31,32,33,34,35] and eukaryotic [17,36,37,38,39] pathogens.

In general, the complex intestinal microbiota of healthy bees plays an irreplaceable role in supporting growth, physiological function, and metabolic processes [40]. It is important to note that not all microorganisms present in this environment can be considered probiotics; true probiotics must meet specific criteria and requirements [41].

Several host-adapted probiotic strains have been shown to inhibit *Paenibacillus larvae*, the etiological agent of American foulbrood, through distinct antimicrobial mechanisms. For instance, *Lactobacillus kunkeei* strains isolated from the honey bee crop have demonstrated significant inhibitory effects against *P. larvae* in vitro, attributed to their production of hydrogen peroxide and organic acids [42]. Likewise, *Bifidobacterium asteroides* and *Lactobacillus apis* have been reported to suppress *P. larvae* growth and to stimulate host immunity in bee larvae, improving survival outcomes during infection [30,43]. Despite these findings, most studies have focused on limited isolate diversity, leaving the inhibition profiles of broader clinical strains largely unexplored. Addressing this gap, the present study investigates the antagonistic potential of 111 *Lactobacillus* and *Bifidobacterium* strains, originating from the digestive tracts of honey bees collected across eight regions in the Czech Republic, against four clinical *P. larvae* isolates, *Melissococcus plutonius*, and the opportunistic pathogen *Serratia marcescens* strain sicaria, using a modified BHI co-cultivation approach.

## 2. Materials and Methods

### 2.1. Materials

Modified Brain Heart Infusion (mBHI) broth was used as the primary enrichment medium for the isolation of gut-associated bacteria. The composition of the mBHI broth per liter included the following: BHI (Carl Roth, Karlsruhe, Germany) 37 g, glucose 8 g, yeast extract (Oxoid, Hampshire, UK) 3 g, soybean peptone 3 g, meat extract (Carl Roth, Germany) 2 g, KH_2_PO_4_ 2 g, MgCl_2_ 0.5 g, and 0.5 mL of Tween 80 (Thermo Fisher Scientific, Waltham, MA, USA). The pH was adjusted to 7.1–7.3 using 10 M NaOH. Anaerobic conditions were established by CO_2_ treatment. For selective bacterial isolation, three types of agar media were used: mBHI agar supplemented with mupirocin (100 mg/L) and acetic acid (1 mL/L) for selective Bifidobacterium isolation [44], Rogosa agar (Oxoid, UK) for *Lactobacillus*, and MRS agar (Oxoid, UK) to enhance the recovery of other lactic acid bacteria. Anaerobic incubation was achieved using AnaeroGen 3.5 L sachets (Oxoid, UK) in 3.5 L anaerobic jars (Helago-CZ, Prague, Czech Republic). All pure isolates were preserved at −85 °C in 20% (*v/v*) anaerobic glycerol stocks buffered with 1.2 g/L K_2_HPO_4_ and 0.33 g/L KH_2_PO_4_, pH: 6.8–7.4.

### 2.2. Isolation of Bifidobacterium and Lactobacillus Strains

Worker honey bees were sampled from eight different locations in the Czech Republic (Table 1), with 3–5 individuals collected per site. The bees were anesthetized using CO_2_ and dissected aseptically with sterile forceps. The entire gut contents from each group (weighing 80–200 mg in total) were pooled and transferred into Hungate anaerobic tubes containing 1.8 mL of sterile mBHI broth. Serial dilutions of the gut homogenates were prepared in anaerobic conditions, and aliquots of 0.05–0.2 mL from suitable dilutions were plated on the selective agar media. Plates were incubated at 37 °C for 72 h under anaerobic conditions. Colonies with different morphologies were selected, subcultured in anaerobic mBHI or MRS broth, and preserved for further analysis.

### 2.3. Isolation of Bacterial Honey Bee Pathogens

Four *Paenibacillus larvae* strains—PL2, PL41, PL48, and PL52—were provided by the Czech Bee Research Institute (CBRI) in Dol–Máslovice, Czech Republic. Strains PL2, PL48, and PL52 were isolated from larvae exhibiting clinical symptoms of American foulbrood, while strain PL41 was isolated from hive debris. These strains originated from four localities within the Czech Republic: PL2—Brejl, Rakovník (GPS: 50°6′8.248″ N, 13°52′23.312″ E); PL41—Lípa-Paseky (49°11′35.859″ N, 17°46′48.737″ E); PL48—Římovice (49°41′46.842″ N, 14°56′34.802″ E); and PL52—Valtířov (50°40′25.339″ N, 14°7′32.343″ E). Bacterial strains were isolated using the microbiological cultivation technique described previously [45].

The *Melissococcus plutonius* strain CZ2020, isolated according to the protocol of Thebeau et al. [46], was also supplied by the CBRI for research purposes.

Professor James B. Burritt of the University of Wisconsin–Stout kindly provided the *Serratia marcescens* strain sicaria, an opportunistic honey bee pathogen [47].

### 2.4. Classification of Isolates

Overnight cultures (1 mL) grown in mBHI medium were used for chromosomal DNA extraction using the PrepMan Ultra Sample Preparation Reagent (Thermo Fisher Scientific, USA). Diluted DNA samples, ranging from 1:100 to 1:10,000 and reaching concentrations of 20–120 ng/μL, were used for 16S rDNA amplification. PCR mixtures (30 μL) consisted of 1× EliZyme™ FAST Taq MIX Red (Elisabeth Pharmacon, Brno-Zidenice, Czech Republic), 0.5 μM of each primer, PCR-grade ultra-pure H_2_O, and template DNA at a concentration of 10–100 ng. PCR was performed in a TProfessional Gradient 96 thermocycler (Biometra, Göttingen, Germany) under the following conditions: initial denaturation at 94 °C for 5 min; 36 cycles of denaturation at 94 °C for 1 min, annealing at 49 °C for 100 s, and extension at 72 °C for 2 min; followed by a final extension step at 72 °C for 7 min. PCR amplification was carried out using primer pairs FP1–RP2 [48] or 27F–1542R [49]. Amplicons (~1500 bp) were checked via 1.5% agarose gel electrophoresis and purified using the Monarch PCR & DNA Cleanup Kit (New England Biolabs, Ipswich, MA, USA). The purified products were then bidirectionally sequenced by SEQme (Brno, Czech Republic). Assembled sequences, ranging from 1332 to 1481 nucleotides, were processed in Geneious v7.1.7 (Biomatters Ltd., Auckland, New Zealand) and deposited in the NCBI database using the BankIt submission tool. Taxonomic classification was performed based on 16S rDNA sequence pairwise identity with the closest related taxa using the EzBioCloud database [50]. The same procedure was applied for the classification of the four *P. larvae* clinical isolates, *M. plutonius*, and *Serratia marcescens* strains.

### 2.5. In Vitro Inhibition of Honey Bee Bacterial Pathogens

A total of one hundred eleven bacterial strains (Table 1) were selected for in vitro assays. All tested strains, including bee (opportunistic) pathogens, were capable of growing in mBHI medium (as described above) under anaerobic conditions. Therefore, this common growth medium, supplemented with bacteriological agar (14 g/L), was used for the inhibition testing. Pathogenic cultures revived in mBHI broth and harvested at the late logarithmic phase were applied in a volume of 0.2 mL (achieving a cell concentration of 6.08–7.4 log CFU—colony-forming units) onto Petri dishes (90 mm in diameter). The inoculated cultures were then overlaid with 20 mL of mBHI agar. After solidification, 0.08 mL of 14–18 h-old bacterial cultures, also in the late logarithmic phase [51] and reaching a concentration of 6.7–7.2 log CFU, was applied to a central well (radius 10 mm). The co-culture samples were incubated under microaerophilic conditions (CampyGen 3.5L; Oxoid, UK) in 3.5 L anaerobic jars (Helago-CZ, Czech Republic) for 120 h at 37 °C. After incubation, the radii of the lytic zones (in mm) were measured. Samples that exhibited inhibition zones were subsequently tested in triplicate. All samples were visually compared with pure cultures of bee pathogens grown under identical conditions.

Selected samples exhibiting inhibition zones were documented by microphotography for publication purposes.

### 2.6. Phylogenetic Analyses

To evaluate the evolutionary relationships between active and inactive honey bee-associated *Bifidobacterium* and *Lactobacillus* strains, phylogenetic trees based on 16S rRNA gene sequences were reconstructed using MEGA version 5.05 and the neighbor-joining method [52]. The Kimura 2-parameter model was applied for the tree construction. All strain types of currently described *Bifidobacterium* and *Lactobacillus* species known to exclusively inhabit the honey bee digestive tract were included in the phylogenetic analyses. Their 16S rRNA gene sequences were retrieved from the NCBI nucleotide database (https://www.ncbi.nlm.nih.gov/nuccore/). Particular gene codes are written in parentheses next to specific taxa in the phylogenetic trees.

## 3. Results

### 3.1. Classification of Bacterial Strains

Table 1 presents the classification of bacterial strains based on 16S rDNA sequence identity. GenBank accession numbers for the 16S ribosomal RNA genes are also provided. Strains sharing ≥99.4% identity with the closest type strains were classified at the species level. Strains showing ≤98.7% identity [53] were designated by their respective generic names and marked as p.n.sp. (putative new species). Almost all known host-specific species of honey bee-associated *Bifidobacterium* with the exception of *Bifidobacterium apis*—were isolated from captured bees [9]. These included the following: *B. apousia* (2 strains), *B. asteroides* (12), *B. choladohabitans* (7), *B. mellis* (2), *B. mizhiense* (1), and *B. polysaccharolyticum* (11). Fourteen additional *Bifidobacterium* strains may represent potentially novel species. Most host-specific *Lactobacillus* species (excluding members of the Firm-5 phylotype group, which belongs to the genus *Bombilactobacillus*) inhabiting the digestive tract of European honey bees were identified as follows: *Lactobacillus apis* (16 strains), *L. helsingborgensis* (14), *L. kullabergensis* (4), *L. kimbladii* (4), and *L. melliventris* (20) [8,54]. Two strains are considered potentially novel species within the genus *Lactobacillus*.

The 16S rRNA gene sequences of clinical isolates PL2, PL41, and PL48 were deposited in the NCBI database under GenBank accession numbers OR050945, OR050947, and OR050948, respectively. These strains, along with PL52, share identical sequences and exhibit 99.86% sequence similarity to *Paenibacillus larvae* ATCC 9545^T^. The 16S rRNA gene sequence (1450 bp) of *Melissococcus plutonius* CZ2020 displayed 100% identity with *M. plutonius* ATCC 35311^T^. Similarly, the taxonomic identity of *Serratia marcescens* strain sicaria (Ss1) was confirmed via comparative analysis [47].

### 3.2. In Vitro Inhibition of Honey Bee Bacterial Pathogens

Results are presented in Table 1. Inhibitory activity was assessed based on the diameter of inhibition zones and categorized as follows: weak inhibition (16–20 mm), moderate inhibition (21–24 mm), high inhibition (25–30 mm), and strong inhibition (>30 mm). A total of 29 strains (~26%), including 15 *Bifidobacterium* and 14 *Lactobacillus* strains, exhibited at least moderate inhibitory activity against two clinical *P. larvae* isolates. Among these, 12 strains (6 *Bifidobacterium* and 6 *Lactobacillus*; ~12%) inhibited all 4 *P. larvae* clinical strains to some extent. Only 6 strains—*Bifidobacterium choladohabitans* (2 strains), *Bifidobacterium* sp. (2), *Lactobacillus apis* (1), and *L. helsingborgensis* (1)—demonstrated at least moderate inhibition against 2 or more *P. larvae* strains as well as *S. marcescens* strain sicaria. The strongest inhibitory effects against *P. larvae* were observed in strains belonging to *Bifidobacterium asteroides*, *B. polysaccharolyticum*, *Bifidobacterium* sp., *L. helsingborgensis*, *L. apis*, *L. kullabergensis*, and *L. melliventris.*

A total of 20 strains (~18%) showed inhibitory activity against the *S. marcescens* strain sicaria. Of these, 12 strains (8 *Bifidobacterium*, 4 *Lactobacillus*) produced at least moderate inhibition. In contrast, *M. plutonius* CZ2020 was minimally inhibited, with only three strains forming visible inhibition zones. The most active strains overall were affiliated with *Bifidobacterium asteroides*, *B. polysaccharolyticum*, *B. choladohabitans*, *Bifidobacterium* sp., *Lactobacillus apis*, and *L. helsingborgensis* (see Table 1).

**Figure 1:**
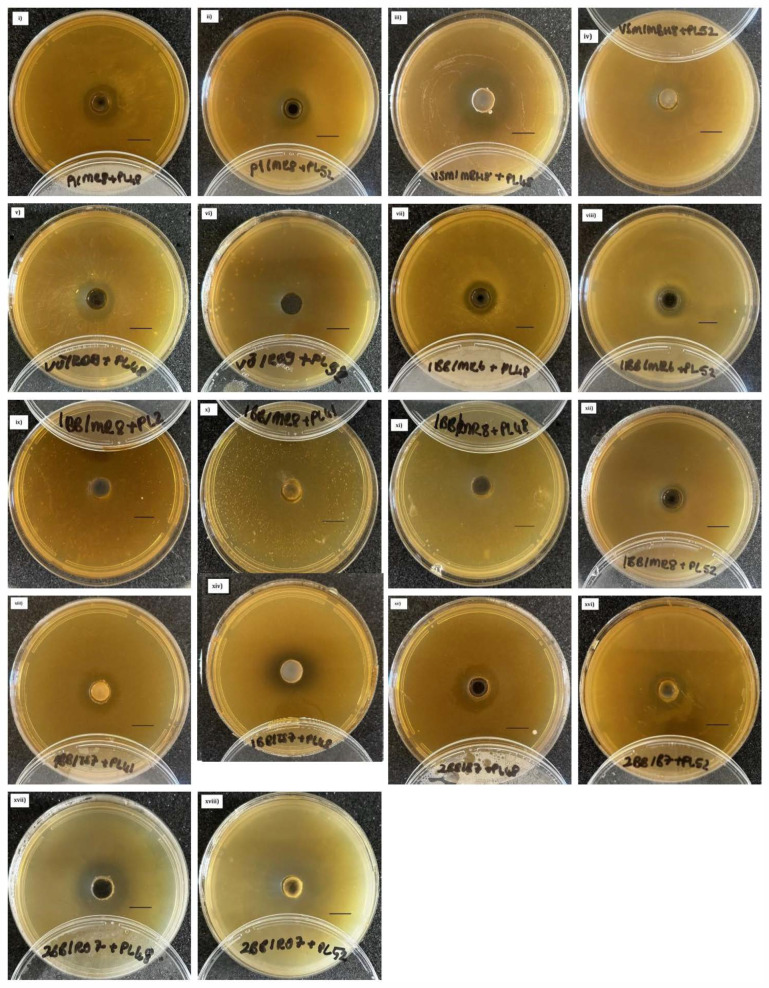
Examples of inhibition zones observed in co-cultures of selected *Lactobacillus* and *Bifidobacterium* strains with *Paenibacillus larvae* clinical isolates. Shown combinations include: (**i**) *Lactobacillus apis* P1/MR8 + PL48; (**ii**) *L. apis* P1/MR8 + PL52; (**iii**) *Bifidobacterium choladohabitans* VSM/mBH8 + PL48; (**iv**) *B. choladohabitans* VSM/mBH8 + PL52; (**v**) *L. melliventris* VU/RO9 + PL48; (**vi**) *L. melliventris* VU/RO9 + PL52; (**vii**) *L. apis* 1BB/MR6 + PL48; (**viii**) *L. apis* 1BB/MR6 + PL52; (**ix**) *L. apis* 1BB/MR8 + PL2; (**x**) *L. apis* 1BB/MR8 + PL41; (**xi**) *L. apis* 1BB/MR8 + PL48; (**xii**) *L. apis* 1BB/MR8 + PL52; (**xiii**) *Bifidobacterium* sp. 1BB/TS7 + PL41; (**xiv**) *Bifidobacterium* sp. 1BB/TS7 + PL48; (**xv**) *B. choladohabitans* 2BB/B7 + PL48; (**xvi**) *B. choladohabitans* 2BB/B7 + PL52; (**xvii**) *L. melliventris* 2BB/RO7 + PL48; (**xviii**) *L. melliventris* 2BB/RO7 + PL52. Scale bar: 10 mm.

**Figure 2:**
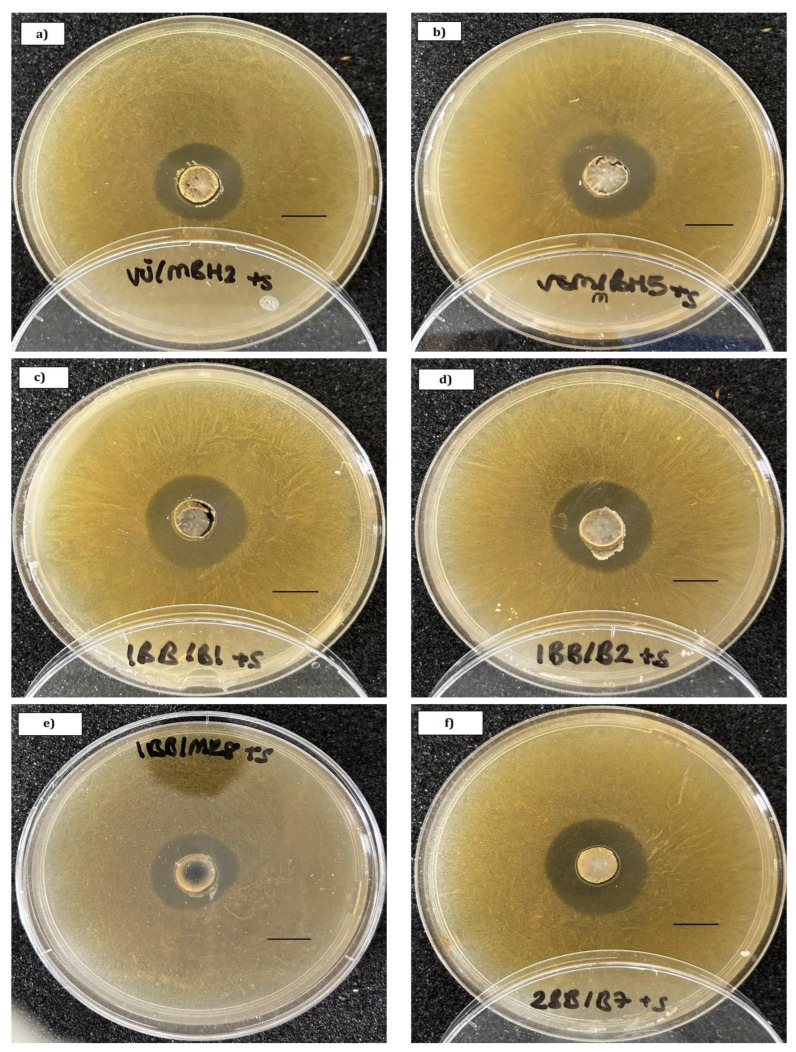
Examples of inhibition zones in co-cultures of *Serratia marcescens* strain sicaria with (**a**) *B. polysaccharolyticum* VU/mBH2, (**b**) *B. polysaccharolyticum* VSM/mBH5, (**c**) *Bifidobacterium* sp. 1BB/B1, (**d**) *Bifidobacterium* sp. 1BB/B2, (**e**) *L. apis* 1BB/MR8, (**f**) *B. choladohabitans* 2BB/B7. Scale bar: 10 mm.

Representative microphotographs of inhibition zones against *P. larvae* and *S. marcescens* are shown in Figure 1 and Figure 2, respectively.

### 3.3. Phylogenetic Analyses

**Figure 3:**
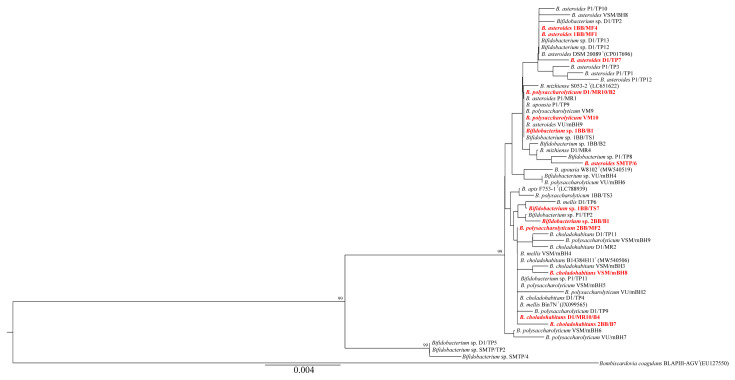
Phylogenetic tree reconstructed based on the 16S rRNA gene (length: 1236 nt), including bifidobacterial strains from this study and type strains of Bifidobacterium taxa originating exclusively from honey bees. GenBank accession codes for these strains are given in parentheses. Additional GenBank accession numbers are listed in Table 1. Bootstrap values ≥ 70%, based on 1000 replicates, are shown at the nodes. Scale bar: 0.004 substitutions per nucleotide position.

**Figure 4:**
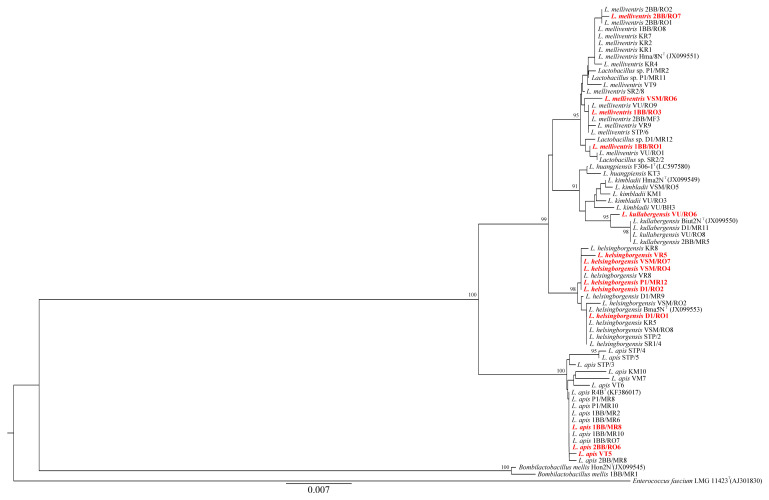
Phylogenetic simulation based on the 16S rRNA gene (length: 1330 nt), showing *Lactobacillus* strains from this study and type strains of *Lactobacillus* taxa previously isolated exclusively from honey bees. GenBank accession numbers for these strains are given in parentheses; additional accession numbers are listed in Table 1. Bootstrap values ≥ 70%, based on 1000 replicates, are shown at the nodes. Scale bar: 0.007 substitutions per nucleotide position.

*Bifidobacterium* and *Lactobacillus* strains exhibiting at least moderate inhibition against at least two *P. larvae* strains are highlighted in red in Figure 3 and Figure 4, respectively. In both cases, active strains are distributed across multiple species, suggesting that inhibitory activity is strongly strain dependent rather than species specific. In the case of *Bifidobacterium* (Figure 3), phylogenetic clusters and branches comprising different species are poorly resolved. This likely reflects the high sequence similarity among *Bifidobacterium* species, as well as the use of a relatively short 16S rRNA gene fragment for tree reconstruction. In contrast, the phylogenetic relationships among *Lactobacillus* strains are more clearly defined (Figure 4), with distinct clusters corresponding to individual species.

## 4. Discussion

In this study, we presented the results of a simple technique that reveals the antagonistic activity of key representatives of the honey bee physiological microbiota against the causative agents of the most serious bacterial infections in bees. This technique is based on the co-cultivation of two taxonomically, physiologically, and metabolically distinct groups of bacteria in a modified BHI medium. *Paenibacillus larvae* strains are characterized as Gram-positive, microaerophilic to facultatively anaerobic, spore-forming, and mostly motile bacteria [55], whereas bifidobacteria and lactobacilli are non-motile, Gram-positive, microaerophilic to anaerobic microorganisms with an obligately fermentative metabolism [56]. *Serratia marcescens*, phylogenetically belonging to the class Gammaproteobacteria, are Gram-negative, rod- to coccoid-shaped, saprophytic, motile, facultatively anaerobic organisms that can colonize a variety of environments and grow over a wide temperature range (5–40 °C) and rely solely on fermentation for energy production, independent of the presence of oxygen [57]. It is considered an opportunistic pathogen of plants, humans, and some insects, including honey bees [47,58]. *Melissococcus plutonius*, the causative agent of European foulbrood, is a Gram-positive, anaerobic, lanceolate coccoid bacterium with a fermentative metabolism, classified within the group of lactic acid bacteria [59]. To support the growth of all bacterial strains used in this study, particularly the nutritionally demanding bifidobacteria and lactobacilli, we fortified the BHI medium (ThermoFisher Scientific, Waltham, MA, USA) commonly used for the cultivation of a wide range of bacteria with glucose, yeast extract, and soy peptone. This approach is unique compared to previous studies, which typically applied separate cultivation of bifidobacteria and lactobacilli [30,31] in the initial phase, followed by co-cultivation with *P. larvae* on different media, mostly MRS broth (agar) and MYPGP agar.

The following taxa exhibited the strongest inhibitory activity against *P. larvae* strains: *Bifidobacterium asteroides*, *B. polysaccharolyticum*, *Bifidobacterium* sp., *Lactobacillus helsingborgensis*, *L. apis*, *L. kullabergensis*, and *L. melliventris* (Table 1). Other studies have identified *Apilactobacillus kunkeei* [30,36,60], *Fructobacillus fructosus* [35], *Lactobacillus apis* [4,33], *L. panisapium* [33], *L. melliventris* [30,33], *L. kullabergensis* [30,33], *L. kimbladii* [30,33], *L. mellis* [33], and unclassified bifidobacteria from the *B. asteroides* group [30,33] as active taxa against *P. larvae*. Compared to these studies, we tested a broader spectrum of bifidobacterial and lactobacilli strains against four different clinical isolates of *P. larvae*. Only one previous study used four genetically distinct *P. larvae* strains for similar purposes. Forsgren et al. [30] also observed that only some of the 11 bifidobacterial and lactobacilli strains tested exhibited antagonistic effects on all four clinical isolates. Inhibitory activity against *P. larvae* was first demonstrated in various *Lactobacillus helsingborgensis* strains (notably D1/RO1, D1/RO2, P1/MR12, VR5, VSM/RO4; see Table 1), and especially in particular strains of *Bifidobacterium asteroides*, *B. polysaccharolyticum*, and *Bifidobacterium* sp. Notably, the strains designated as *Bifidobacterium* sp. are likely to represent novel species. The inhibitory effect was strongly strain dependent, as active strains were distributed across different regions of the phylogenetic trees (Figure 3 and Figure 4) and among distinct species clusters. These findings are consistent with the high intra-phylotype diversity observed among lactobacilli and bifidobacteria inhabiting the honey bee gut [54]. The specific bioactive compounds produced by bifidobacteria and lactobacilli responsible for antagonistic effects against *P. larvae* and other bacterial pathogens remain largely unexplored. Compounds such as heptyl 2-methylbutyrate, di-isobutyl phthalate, heptakis (trimethylsilyl), di-isooctyl phthalate, and hyodeoxycholic acid have been detected in metabolites of *Fructobacillus fructosus* active against *P. larvae* [35]. Butler et al. [61] speculated that extracellular proteins of lactic acid bacteria, including bacteriocins and lysozymes, may be the primary antimicrobials in honey bees.

In contrast to previous studies that demonstrated antimicrobial activity of various bifidobacteria [34] and lactobacilli [31,43] against *M. plutonius*, we observed at most weak inhibition in only three strains (Table 1). This discrepancy may be due to methodological differences and the use of different culture media and also to strain-specific characteristics of the pathogen. Recent genomic analyses have revealed significant heterogeneity among *M. plutonius* isolates, affecting their susceptibility to antimicrobial agents [62]. Furthermore, environmental conditions such as pH and nutrient availability can influence the production of antimicrobial metabolites by probiotics [63]. For instance, the lack of certain growth-promoting factors under our experimental conditions may have hindered bacteriocin or organic acid production. Finally, increasing reports of antibiotic resistance in *M. plutonius*, such as Masood et al. [62], suggest that resistance mechanisms may also diminish the efficacy of probiotic interventions. These factors should be considered in future in vitro and in vivo evaluations.

*Serratia marcescens* is a Gram-negative opportunistic pathogen known to infect a broad spectrum of hosts, including both insects and humans. In honey bees, it has been primarily associated with diseased larvae and only sporadically detected in adult bees, often at very low abundances, which is considered indicative of an imbalanced gut microbiota. Recent studies have reported that certain strains of *S. marcescens*, such as those isolated from the bee gut or Varroa mites, can increase mortality in adult bees, particularly following exposure to antibiotics or pesticides [58]. Research focusing on the inhibitory effects of the bee gut microbiota against virulent strains of *S. marcescens* is scarce. One relevant study by Lang et al. [32] reported that *Gilliamella apicola* and *Lactobacillus apis* protect honey bees from this opportunistic pathogen. The study by Chen et al. [2] focused on the taxonomic classification and host specificity of *B. choladohabitans* and did not address its potential probiotic activity against bee pathogens. In our present study, certain *B. choladohabitans* strains showed remarkable inhibitory effects against important pathogens, especially *Paenibacillus larvae*. This finding suggests a new functional role for this species and significantly expands the current knowledge about honey bee gut microbiota. In this context, the demonstration of antimicrobial activity of *B. choladohabitans* highlights its importance as a promising candidate for future probiotic applications. Our study presents the first results on the inhibitory activity of bifidobacteria and lactobacilli against *S. marcescens* strain sicaria. Since this is a Gram-negative bacterium, active strains, especially *B. asteroides*, *B. polysaccharolyticum*, *B. choladohabitans*, *Bifidobacterium* sp., and *Lactobacillus apis* (Table 1), must produce different antimicrobial substances (e.g., H_2_O_2_, diacetyl) compared to those effective against Gram-positive pathogens.

It is worth noting that the observed results may be influenced by the methodology. In some cases, the inhibition zones were not sharply defined (e.g., Figure 1: images vi, ix, x, xiv), which may have been caused by the diffusion of antimicrobial compounds throughout the agar medium. Additional factors such as species- or strain-specific growth characteristics, culture medium composition, atmospheric conditions, and temperature could also affect the in vitro inhibitory effects.

The commercial use of probiotics in honey bees is a developing field of research with the aim of improving colony health and productivity and reducing dependence on chemical treatments. Probiotics from the Lactobacillus and Bifidobacterium species are known for their beneficial effects on intestinal health, immune system strengthening, and pathogen suppression properties and have also shown positive results in honey bees [64,65]. Various studies have shown that probiotic strains can modulate the gut microbiota of honey bees and increase their resistance to diseases such as *Paenibacillus larvae* and *Melissococcus plutonius* [60,64,66].

In order for probiotics to be used commercially in beekeeping, they must meet certain criteria such as safety, efficacy, and ability to survive in hive conditions. These probiotics must be free of virulence factors that may have adverse effects on honey bee health [67]. They must also be resistant to environmental stressors such as temperature changes and pesticides. Probiotics are expected to effectively colonize bee guts, enhance their antimicrobial properties, and support overall colony health [60,67]. Probiotics may offer a variety of benefits beyond disease prevention. Studies have shown that probiotics can increase the nutrient digestion capacity of honey bees and also increase their resistance to pesticides and malnutrition [65]. These benefits suggest that probiotics may be a sustainable alternative to antibiotics and other chemical treatments, which are often associated with negative ecological consequences such as resistance development.

This study emphasized the concept of strain-dependent inhibition to assess the antimicrobial potential of *Lactobacillus* and *Bifidobacterium* strains against major honey bee pathogens, with the broader goal of supporting their potential commercial application as probiotics. The relevance of strain-dependent inhibition lies in its critical role in selecting effective probiotic candidates for honey bee health management. Our results demonstrate that only a fraction of the tested strains exhibited strong and wide-ranging inhibitory effects against multiple pathogenic bacteria. This finding underscores that antimicrobial activity is not consistent at the species level but instead varies significantly between individual strains. Understanding this strain-specific variability is essential for the rational selection of probiotics aimed at enhancing colony resilience against bacterial infections, including foulbrood diseases and opportunistic pathogens like *Serratia marcescens*. However, more research is needed before probiotics can be widely accepted in beekeeping. Future studies should evaluate the efficacy of probiotics in real-world conditions and examine their long-term effects on hive productivity, colony growth, and survival.

Overall, the antagonistic properties of specific bacterial strains should be experimentally verified in both laboratory and field settings using honey bees infected with relevant pathogens. In future research, the most active strains should also be assessed for additional beneficial effects, such as their potential impact on host immune and metabolic functions. Furthermore, strains with uncertain taxonomic status (e.g., *Bifidobacterium* sp.) should be taxonomically classified in detail using modern phylogenomic, chemotaxonomic, and biochemical approaches.

## 5. Conclusions

The results of our study confirmed, as reported in many previous investigations, that the antimicrobial properties of probiotic bacteria are strongly strain dependent. A strong antagonistic effect against *P. larvae* strains was observed particularly in strains of *Bifidobacterium asteroides* (~36% of strains showed moderate inhibition of at least two *P. larvae* strains), *B. polysaccharolyticum* (~36%), *Bifidobacterium* sp. (~31%), *Lactobacillus helsingborgensis* (~43%), L. apis (~19%), *L. kullabergensis* (~33%), and *L. melliventris* (20%). Only the strains *Bifidobacterium choladohabitans* VSM/mBH8 and 2BB/B7; *Bifidobacterium* sp. 1BB/B1 and 1BB/B2; *Lactobacillus apis* 1BB/MR8; and *L. helsingborgensis* VSM/RO7 showed at least moderate inhibition of two or more *P. larvae* strains and also of *S. marcescens*.

This is the first study to report on the antimicrobial activity of bifidobacteria and lactobacilli against the *Serratia marcescens* strain sicaria. The strongest inhibitory effect was observed in strains of *Bifidobacterium asteroides*, *B. polysaccharolyticum*, *B. choladohabitans*, *Bifidobacterium* sp., *Lactobacillus apis*, and *L. helsingborgensis*.

The simple in vitro technique presented here offers a practical and efficient method for screening a broad range of honey bee symbionts against various bacterial pathogens of honey bees. Antagonistic activity against different honey bee pathogens is a crucial probiotic property. In this context, we recommend testing a diverse collection of isolates representing the physiological symbiotic microbiota of the honey bee gut, obtained from different geographic locations, against multiple clinical isolates of *P. larvae* and other pathogenic or opportunistically pathogenic microorganisms affecting honey bees.

In addition to screening for antimicrobial activity, future studies should also evaluate other probiotic functions such as enzyme production and xenobiotic degradation potential, particularly for the most active strains identified in this study. These properties are important for understanding the broader potential of these probiotics to support honey bee health and promote colony resilience, as they may provide valuable insights into the mechanisms behind the observed antagonistic effects and may help identify strains with additional beneficial traits for honey bee management.

## Figures and Tables

**Table 1:** Inhibition zones (mm) observed in the tested bacterial strains. Standard deviations from triplicate measurements ranged between 2 and 4 mm. A dash (-) indicates no inhibition (negative result). MP—*Melissococcus plutonius* CZ2020; Ss1—*Serratia marcescens* strain sicaria. The first letter(s) in the strain designation correspond to the locality of origin: D—Dol (Máslovice), GPS: 50°12′14.327″ N, 14°22′0.412″ E; K—Kralupy nad Vltavou, GPS: 50°14′12.379″ N, 14°18′39.844″ E; P—Postřižín, GPS: 50°14′1.426″ N, 14°23′10.259″ E; V—Větrušice, GPS: 50°11′22.276″ N, 14°22′55.871″ E; VSM—Střezimíř, GPS: 49°31′55.215″ N, 14°36′40.781″ E; VU—Červený Újezd, GPS: 49°33′19.444″ N, 14°36′14.613″ E; BB—Roztoky, GPS: 50°9′24.685″ N, 14°23′24.378″ E; S—Suchdol, GPS: 50°7′48.109″ N, 14°22′25.745″ E.

Strain	Classification	PL2	PL41	PL48	PL52	MP	Ss1	GenBank a.n.
D1/TP2	*Bifidobacterium apousia*	-	21	-	-	-	-	PP754609
D1/TP4	*Bifidobacterium choladohabitans*	-	-	-	-	-	-	PP754610
D1/TP5	*Bifidobacterium* sp. (p.n.sp.)	-	-	-	-	-	-	PP754611
D1/TP6	*Bifidobacterium mellis*	-	-	-	-	-	-	PP754612
D1/TP7	*Bifidobacterium asteroides*	-	20	38	38	-	-	PP754613
D1/TP9	*Bifidobacterium asteroides*	-	16	24	-	-	-	PP754614
D1/TP11	*Bifidobacterium choladohabitans*	-	33	-	-	-	-	PP754615
D1/TP12	*Bifidobacterium* sp. (p.n.sp.)	-	-	-	-	-	-	PP754616
D1/TP13	*Bifidobacterium* sp. (p.n.sp.)	-	36	-	-	-	-	PP754617
D1/MR2	*Bifidobacterium choladohabitans*	18	16	20	22	-	-	PP754618
D1/MR4	*Bifidobacterium mizhiense*	-	-	-	-	-	-	PP754619
D1/MR9.	*Lactobacillus helsingborgensis*	-	-	-	36	-	-	PP754620
D1/MR10/B2	*Bifidobacterium polysaccharolyticum*		33	34	42			PP754621
D1/MR10/B4	*Bifidobacterium choladohabitans*	19	25	21	19	-	-	PP754622
D1/MR11	*Lactobacillus kullabergensis*	-	-	-	-	-	-	PP754623
D1/MR12	*Lactobacillus* sp. (p.n.sp.)	-	-	-	-	-	-	PP754624
D1/RO1	*Lactobacillus helsingborgensis*	21	36	-	22	-	-	PP754625
D1/RO2	*Lactobacillus helsingborgensis*	32	22	32	34	-	-	PP754626
KM1	*Lactobacillus kimbladii*	-	-	-	-	-	-	PP754627
KM10	*Lactobacillus apis*	-	-	-	-	-	-	PP754628
KR1	*Lactobacillus melliventris*	-	-	36	22	-	-	PP754629
KR2	*Lactobacillus melliventris*	-	-	-	-	-	-	PP754630
KR4	*Lactobacillus melliventris*	-	-	-	-	-	-	PP754631
KR5	*Lactobacillus helsingborgensis*	20	18	-	24	26	-	PP754632
KR7	*Lactobacillus melliventris*	-	-	-	-	-	-	PP754633
KR8	*Lactobacillus helsingborgensis*	-	-	21	17	-	-	PP754634
KT3	*Lactobacillus huangpiensis*	-	-	-	-	-	-	PP754635
P1/MR1	*Bifidobacterium asteroides*	18	19	18	18	-	21	PP754636
P1/MR2	*Lactobacillus melliventris*	-	17	-	17	-	-	PP754637
P1/MR8	*Lactobacillus apis*	-	24	18	17	-	18	PP754638
P1/MR10	*Lactobacillus apis*	-	31	-	-	17	-	PP754639
P1/MR11	*Lactobacillus melliventris*	-	-	-	-	-	-	PP754640
P1/MR12	*Lactobacillus helsingborgensis*	-	44	34	40	-	-	PP754641
P1/TP1	*Bifidobacterium asteroides*	-	-	21	-	-	-	PP754642
P1/TP2	*Bifidobacterium* sp. (p.n.sp.)	-	-	-	-	-	-	PP754643
P1/TP3	*Bifidobacterium asteroides*	17	18	18	-	-	-	PP754644
P1/TP8	*Bifidobacterium* sp. (p.n.sp.)	-	-	-	-	-	-	PP754645
P1/TP9	*Bifidobacterium apousia*	-	-	-	48	-	-	PP754646
P1/TP10	*Bifidobacterium asteroides*	-	46	-	26	-	-	PP754647
P1/TP11	*Bifidobacterium* sp. (p.n.sp.)	-	-	-	-	-	-	PP754648
P1/TP12	*Bifidobacterium asteroides*	-	-	-	-	-	-	PP754649
VM7	*Lactobacillus apis*	-	-	18	-	-	-	PP754650
VM9	*Bifidobacterium polysaccharolyticum*	19	-	25	20	-	-	PP754651
VM10	*Bifidobacterium polysaccharolyticum*	20	28	21	17	-	-	PP754652
VR5	*Lactobacillus helsingborgensis*	22	22	24	26	22	-	PP754653
VR8	*Lactobacillus helsingborgensis*	-	-	-	-	-	-	PP754654
VR9	*Lactobacillus melliventris*	-	-	-	-	-	-	PP754655
VT5	*Lactobacillus apis*	26	34	26	24	-	-	PP754656
VT6	*Lactobacillus apis*	-	-	-	-	-	-	PP754657
VT9	*Lactobacillus melliventris*	-	-	-	-	-	-	PP754658
VSM/BH8	*Bifidobacterium asteroides*	-	-	-	-	-	-	PP754659
VSM/mBH3	*Bifidobacterium choladohabitans*	-	-	-	-	-	-	PP754660
VSM/mBH4	*Bifidobacterium mellis*	-	-	-	30	-	20	PP754661
VSM/mBH5	*Bifidobacterium polysaccharolyticum*	-	-	-	-	-	24	PP754662
VSM/mBH6	*Bifidobacterium polysaccharolyticum*	-	-	-	-	-	-	PP754663
VSM/mBH8	*Bifidobacterium choladohabitans*	-	-	28	21	-	21	PP754664
VSM/mBH9	*Bifidobacterium polysaccharolyticum*	-	-	-	-	-	23	PP754665
VSM/RO2	*Lactobacillus helsingborgensis*	-	-	-	-	-	-	PP754666
VSM/RO4	*Lactobacillus helsingborgensis*	48	36	52	> 70	-	-	PP754667
VSM/RO5	*Lactobacillus kimbladii*	-	-	-	-	-	-	PP754668
VSM/RO6	*Lactobacillus melliventris*	18	18	24	21	-	-	PP754669
VSM/RO7	*Lactobacillus helsingborgensis*	-	-	21	27	-	23	PP754670
VSM/RO8	*Lactobacillus helsingborgensis*	-	-	-	-	-	-	PP754671
VU/BH3	*Lactobacillus kimbladii*	-	-	-	-	-	-	PP754672
VU/mBH2	*Bifidobacterium polysaccharolyticum*	-	-	-	-	-	21	PP754673
VU/mBH4	*Bifidobacterium* sp. (p.n.sp.)	-	-	-	-	-	20	PP754674
VU/mBH6	*Bifidobacterium polysaccharolyticum*	-	-	-	-	-	18	PP754675
VU/mBH7	*Bifidobacterium polysaccharolyticum*	-	-	-	-	-	-	PP754676
VU/mBH9	*Bifidobacterium asteroides*	-	-	-	-	-	-	PP754677
VU/RO1	*Lactobacillus melliventris*	-	-	-	-	-	-	PP754678
VU/RO3	*Lactobacillus kimbladii*	-	-	-	-	-	-	PP754679
VU/RO6	*Lactobacillus kullabergensis*	36	26	31	51	-	-	PP754680
VU/RO8	*L. kullabergensis*	-	-	-	-	-	18	PP754681
VU/RO9	*L. melliventris*	-	-	20	36	-	-	PP754682
1BB/B1	*Bifidobacterium* sp. (p.n.sp.)	>70	33	26	-	-	25	PP754683
1BB/B2	*Bifidobacterium* sp. (p.n.sp.)	24	-	23	22	-	25	PP754684
1BB/MF1	*Bifidobacterium asteroides*	18	-	25	51	-	19	PP754685
1BB/MF4	*Bifidobacterium asteroides*	24	24	18	-	-	-	PP754686
1BB/MR1	*Bombilactobacillus mellis*	-	-	-	-	-	-	PP754687
1BB/MR2	*Lactobacillus apis*	-	-	27	-	-	-	PP754688
1BB/MR6	*Lactobacillus apis*	-	-	17	17	-	23	PP754689
1BB/MR8	*Lactobacillus apis*	>70	33	30	-	-	22	PP754690
1BB/MR10	*Lactobacillus apis*	-	-	16	16	-	23	PP754691
1BB/RO1	*Lactobacillus melliventris*	39	27	-	24	-	-	PP754692
1BB/RO3	*Lactobacillus melliventris*	>70	26	26	-	-	-	PP754693
1BB/RO7	*Lactobacillus apis*	-	-	-	-	-	18	PP754694
1BB/RO8	*Lactobacillus melliventris*	-	-	-	-	-	-	PP754695
1BB/TS1	*Bifidobacterium* sp. (p.n.sp.)	-	-	-	16	-	-	PP754696
1BB/TS3	*Bifidobacterium polysaccharolyticum*	-	-	-	20	-	-	PP754697
1BB/TS7	*Bifidobacterium* sp. (p.n.sp.)	22	18	29	19	-	-	PP754698
2BB/B1	*Bifidobacterium* sp. (p.n.sp.)	27	46	-	16	-	19	PP754699
2BB/B7	*Bifidobacterium choladohabitans*	22	-	22	20	-	25	PP754700
2BB/MF2	*Bifidobacterium polysaccharolyticum*	-	-	22	26	-	-	PP754701
2BB/MF3	*Lactobacillus melliventris*	-	-	-	20	-	-	PP754702
2BB/MR5	*Lactobacillus kullabergensis*	-	-	-	-	-	-	PP754703
2BB/MR8	*Lactobacillus apis*	-	-	20	18	-	-	PP754704
2BB/RO1	*Lactobacillus melliventris*	-	-	-	-	-	-	PP754705
2BB/RO2	*Lactobacillus melliventris*	-	-	-	-	-	-	PP754706
2BB/RO6	*Lactobacillus apis*	-	-	22	29	-	-	PP754707
2BB/RO7	*Lactobacillus melliventris*	-	-	25	25	-	-	PP754708
SMTP/2	*Bifidobacterium* sp. (p.n.sp.)	-	-	-	-	-	-	PP754709
SMTP/4	*Bifidobacterium* sp. (p.n.sp.)	-	-	-	-	-	-	PP754710
SMTP/6	*Bifidobacterium asteroides*	24	21	17	18	-	-	PP754711
STP/2	*Lactobacillus helsingborgensis*	-	-	-	-	-	-	PP754712
STP/3	*Lactobacillus apis*	-	-	-	-	-	-	PP754713
STP/4	*Lactobacillus apis*	-	-	-	-	-	-	PP754714
STP/5	*Lactobacillus apis*	-	-	-	-	-	-	PP754715
STP/6	*Lactobacillus melliventris*	-	-	-	-	-	-	PP754716
SR1/4	*Lactobacillus helsingborgensis*	-	-	-	-	-	-	PP754717
SR2/2	*Lactobacillus* sp. (p.n.sp.)	-	-	-	16	-	-	PP754718
SR2/8	*Lactobacillus melliventris*	-	-	-	-	-	-	PP754719

## Data Availability

Nearly full-length genes for 16S rRNA of evaluated strains generated by Sanger sequencing are deposited in GenBank under the accession codes PP754609 to PP754719.
